# Driving role of climatic and socioenvironmental factors on human brucellosis in China: machine-learning-based predictive analyses

**DOI:** 10.1186/s40249-023-01087-y

**Published:** 2023-04-12

**Authors:** Hui Chen, Meng-Xuan Lin, Li-Ping Wang, Yin-Xiang Huang, Yao Feng, Li-Qun Fang, Lei Wang, Hong-Bin Song, Li-Gui Wang

**Affiliations:** 1 Center for Disease Control and Prevention of Chinese People’s Liberation Army, 20 Dong-Da-Jie Street, Fengtai District, Beijing, 100071 China; 2 Academy of Military Medical Sciences, Academy of Military Science of Chinese People’s Liberation Army, 27 Taiping Road, Haidian District, Beijing, 100036 China; 3 Chinese Centre for Disease Control and Prevention, No. 155 Changbai Road, Changping District, Beijing, 102206 China; 4 School of Biological Science and Medical Engineering, Beihang University, 37 Xueyuan Road, Haidian District, Beijing, 100191 China; 5 Key Laboratory of Water Cycle and Related Land Surface Processes, Institute of Geographic Sciences and Natural Resources Research, Chinese Academy of Sciences, Beijing, China; 6 State Key Laboratory of Pathogen and Biosecurity, Beijing Institute of Microbiology and Epidemiology, 20 Dong-Da Street, Fengtai District, Beijing, 100071 China

**Keywords:** Human brucellosis, Socioeconomics, Climatic, Extreme weather, Copula model

## Abstract

**Background:**

Brucellosis is a common zoonotic infectious disease in China. This study aimed to investigate the incidence trends of brucellosis in China, construct an optimal prediction model, and analyze the driving role of climatic factors for human brucellosis.

**Methods:**

Using brucellosis incidence, and the socioeconomic and climatic data for 2014–2020 in China, we performed spatiotemporal analyses and calculated correlations with brucellosis incidence in China, developed and compared a series of regression and Seasonal Autoregressive Integrated Moving Average X (SARIMAX) models for brucellosis prediction based on socioeconomic and climatic data, and analyzed the relationship between extreme weather conditions and brucellosis incidence using copula models.

**Results:**

In total, 327,456 brucellosis cases were reported in China in 2014–2020 (monthly average of 3898 cases). The incidence of brucellosis was distinctly seasonal, with a high incidence in spring and summer and an average annual peak in May. The incidence rate was highest in the northern regions’ arid and continental climatic zones (1.88 and 0.47 per million people, respectively) and lowest in the tropics (0.003 per million people). The incidence of brucellosis showed opposite trends of decrease and increase in northern and southern China, respectively, with an overall severe epidemic in northern China. Most regression models using socioeconomic and climatic data cannot predict brucellosis incidence. The SARIMAX model was suitable for brucellosis prediction. There were significant negative correlations between the proportion of extreme weather values for both high sunshine and high humidity and the incidence of brucellosis as follows: high sunshine, $r$ = −0.59 and −0.69 in arid and temperate zones; high humidity, $r$ = −0.62, −0.64, and −0.65 in arid, temperate, and tropical zones.

**Conclusions:**

Significant seasonal and climatic zone differences were observed for brucellosis incidence in China. Sunlight, humidity, and wind speed significantly influenced brucellosis. The SARIMAX model performed better for brucellosis prediction than did the regression model. Notably, high sunshine and humidity values in extreme weather conditions negatively affect brucellosis. Brucellosis should be managed according to the “One Health” concept.

**Supplementary Information:**

The online version contains supplementary material available at 10.1186/s40249-023-01087-y.

## Background

Brucellosis, caused by *Brucella*, remains one of the most common zoonotic diseases worldwide. [1]. In recent years, the incidence rate of human brucellosis (HB) has rapidly increased [2, 3]. HB is usually associated with direct contact with infected livestock or ingestion of unpasteurized dairy products from infected animals [4]. Brucellosis has remained a major public health problem in China [5, 6]. Since the 1990s, the incidence rate of brucellosis has been increasing, and it has been listed as one of the ten most common class A and class B infectious diseases in the People’s Republic of China according to the national legislation for the prevention and control of infectious diseases [2]. According to the latest literature, from 1950 to 2018, the national infectious disease surveillance system in China reported 6,84,380 HB cases [7]. The incidence of HB peaked in 2014 (4.32/100,000), and the geographical range from historically affected northern China to the southern provinces significantly expanded [8, 9]. The National Brucellosis Prevention and Control Plan (NBPCP; 2016–2020) was framed to prevent and control brucellosis [10]. After the implementation of the plan, the serum prevalence of brucellosis among high-risk occupational groups in some areas decreased significantly, although more data are needed for a comprehensive evaluation.

In recent years, studies have shown that global warming has increased the activity range of animals that carry viruses, increased the transmission probability of zoonoses, and has become one of the main reasons for zoonotic transmission [11, 12]. However, the impact of climate change on zoonosis, especially brucellosis, has been largely ignored [13]. By analyzing the relationship between the distribution of HB in China and socioeconomic, environmental, and ecological factors from 2004 to 2017, Peng et al. reported a significant correlation between gross domestic product (GDP), climate, and brucellosis cases herein [14]. Cao et al. used the autoregressive integrated moving average (ARIMA) model to prove that atmospheric pressure, wind speed, mean temperature, and relative humidity significantly impacted brucellosis [15]. Liu et al. used a distributed lag nonlinear model to show that changes in climatic factors, especially changes in temperature, sunshine hours, and evaporation, significantly influence seasonal fluctuations of HB [16]. Other studies have shown that brucellosis is strongly correlated with the normalized difference vegetation index (NDVI) and the numbers of cattle and sheep [17, 18]. However, these studies have mainly focused on areas with a high incidence of brucellosis and the research results are limited to the correlation analysis between economic and climate factors and HB. Thus, there is a lack of in-depth and comprehensive analysis of the factors influencing HB in China.

Climate change poses a greater challenge to preventing and controlling brucellosis in China [19, 20]. At present, research analyzing the temporal and spatial patterns of brucellosis in China using high-quality national incidence rate data is lacking. We describe the scale and distribution of brucellosis in China and emphasize the recent recurrence by analyzing data from city-level monthly reported cases of HB in China from 2014 to 2020. To further understand this mechanism, we used relevant climatic and socioeconomic data to analyze the main influencing factors of HB by building a mathematical model, which will help promote the monitoring and early warning of brucellosis outbreaks.

## Methods

### Data collection and study area

We obtained climate data, including precipitation, sunshine duration, relative humidity, wind speed, and temperature for over 300 prefecture-level cities in China from the China Climatic Data Sharing Service System [21]. Urban socioenvironmental data were obtained from the city statistical yearbook [22], and the incidence data of HB were obtained from the Data Center for China Public Health Science [23]. This study focused on climatic, socioenvironmental, and brucellosis data for Chinese prefecture-level cities, from 2014 to 2020. Therefore, we obtained various types of data during these 7 years.

### Data preprocessing and classification

High data resolution exponentially increases computational complexity, whereas low resolution leads to unclear trends in results and a lack of statistical significance. We used average monthly climatic and brucellosis data to balance the model’s performance and accuracy. The data mainly included monthly average precipitation (MAP), monthly average sunshine (MAS), monthly average humidity (MAH), monthly average wind speed (MAWS), monthly average temperature (MAT), and monthly average incidence (MAI). The raw socioenvironmental data were annual compilations; therefore, it was impossible to perform monthly average processing analysis. We performed a fundamental statistical analysis of all data before formal modeling using SPSS Statistics version 28 (SPSS Inc., Chicago, USA).

There are various methods of geographic zoning in China; in this study, we used traditional north–south zoning for climatic conditions and economic conditions. North–south zoning, with the Qinling Mountains-Huaihe River line as the dividing line, is China’s most common and accepted zoning method [14]. Specifically, China can be divided into five major climatic zones based on the Köppen climate [24, 25]: equatorial, arid, warm, cold, temperate, and polar, and four major geographical regions based on economic conditions: east, central, west, and northeast [26]. To correlate the results with meteorology and socioenvironmental science, we divided the Chinese prefecture-level cities used in this study into economic and climatic zone regions according to the above general guidelines.

Furthermore, this study focused on the effects of weather extremes on the incidence of brucellosis in China. There are many ways to select and define extreme weather conditions based on different criteria. We set the quantile threshold through comparative analysis as a suitable extreme weather classification for our data [27]. For marginal distributions of selected climatic data, we defined values less than one-quarter or more than three-quarters of the range as extreme weather intervals.

### Model overview

This study used several prediction models for climatic, socioenvironmental, and brucellosis data for comparative analysis, starting with classical statistical regression models. Based on the nature of the data and prior statistical analysis results, we used stepwise regression, ridge regression, robust regression, quantile regression, and partial least squares (PLS) regression after model selection. In these regression models, quantitative climatic and socio-environmental data, and qualitative regional classification data were used as independent variables, and brucellosis data were used as dependent variables for the input and output of the results.

Moreover, we improved the machine learning model using a seasonal autoregressive integrated moving average with exogenous variables (SARIMAX) dedicated to time-series prediction. Compared to the SARIMA model for seasonal time-series prediction, the SARIMAX model is mainly suitable for studying the effects of exogenous variables on seasonal time-series prediction and is typically used in climatic prediction studies. Compared to the parameters $p$, $d$, and $q$ of the classical ARIMA model, the SARIMAX model includes four new parameters, namely $P$ (seasonal autoregressive order), $D$ (seasonal difference order), $Q$ (seasonal moving average order), and $S$ (seasonal cycle step). This study used data on various climatic conditions as exogenous variables. The model selection criteria for SARIMAX were the Akaike information criterion (AIC), Bayesian information criterion (BIC), and Hannan-Quin information criterion (HQIC) for assessing information loss.

Finally, we used a copula model to eliminate collinearity between climatic data and analyze extreme weather's effect on brucellosis. We used three marginal distributions (Weibull, Gumbel, and Frechet) and three copula functions (Frank, Gumbel, and Clayton) to analyze the two types of climatic data with the highest absolute values of Kendall correlation coefficients with brucellosis to screen for the best performing model. The filtering criterion for the edge distribution was the goodness-of-fit (GOF) ${R}^{2}$ maximum. In contrast, the filtering criterion for the copula function is AIC.

The copula function is a statistical theory that quantifies the correlation between random variables [28, 29], and its core connection formula is as follows:

$$F\left({X}_{1},{X}_{2}\right)=C\left({F}_{1}\left({X}_{1}\right),{F}_{2}\left({X}_{2}\right)\right)$$

where $F$ is the joint probability density function; $C$ is the copula function; and ${F}_{1}$ and ${F}_{2}$ are the marginal cumulative distribution functions of the two random variables. The domain of the copula function $C$ is defined on an N-dimensional space of $\left[\mathrm{0,1}\right]$, and a monotonically increasing function in each dimension. Boundary conditions must satisfy the following equations:

$$C\left(u,0\right)= C\left(0,v\right)=0,$$

$$C\left(u,1\right)= C\left(1,u\right)=u,$$

$$C\left(v,1\right)= C\left(1,v\right)=v$$

In addition, any point on the copula function c must fulfill the following inequality:

$$C\left({u}_{1},{u}_{2}\right)+ C\left({v}_{1},{v}_{2}\right)-C\left({u}_{1},{v}_{2}\right)- C\left({u}_{2},{v}_{1}\right)\ge 0$$

The formula for the two-dimensional parametric copula function applicable to the climatic data used in this study is as follows:

Frank copula

$${C}_{\theta }^{F}\left(u\right)= -\frac{1}{\theta }\mathrm{log}\left(1+\frac{\left(exp\left(-\theta {u}_{1}\right)-1\right)\left(exp\left(-\theta {u}_{2}\right)-1\right)}{\mathit{exp}\left(-\theta \right)-1}\right), u\in {[\mathrm{0,1}]}^{2}$$

Gumbel–Hougaard copula (in two-dimensional state)

$${C}_{\theta }^{GH}\left(u\right)= exp\left(-{\left(\sum_{j=1}^{2}{\left(-\mathrm{log}{u}_{j}\right)}^{\theta }\right)}^{\frac{1}{\theta }}\right),u\in {[\mathrm{0,1}]}^{2}$$

Clayton copula (in a two-dimensional state)

$${C}_{\theta }^{C}\left(u\right)= \mathit{max}{\left\{{u}_{1}^{-\theta }+{u}_{2}^{-\theta }-1, 0\right\}}^{-\frac{1}{\theta }},u\in {[\mathrm{0,1}]}^{2}$$

where ${u}_{1}$ and ${u}_{2}$ represent two random variables.

## Results

### Spatial and temporal distributions of human brucellosis

From 2014 to 2020, 327,456 HB cases were reported in China. In general, the incidence rate of HB had shown a downward trend since 2014 (57,480 cases, 0.35/100,000 people), with the lowest in 2018, when 37,467 cases (0.22/100,000) were reported. Thereafter, the incidence increased slightly, and 46,884 cases (0.28/100,000) were reported in 2020. From 2014 to 2020, the average annual incidence of brucellosis in the Inner Mongolia Autonomous Region was the highest (3.47/100,000), followed by Ningxia (2.78/100,000), Xinjiang (1.93/100,000), Shanxi (1.13/100,000), and Heilongjiang (1.09/100,000). There are 50 cities with an annual average incidence rate greater than 1/100,000, all of which are in northern China. The incidence rate ranges from 7.71/100,000 in Tacheng, Xinjiang Uygur Autonomous Region, to 1/100,000 in Chengde, Hebei Province. Other cities with high incidence rates include Xing’an League (7.14/100,000), Xilingol League (6.13/100,000) and Tongliao City (5.50/100,000) in Inner Mongolia, Hami City (5.62/100,000) in Xinjiang, Altay Region (5.27/100,000) and Changji Hui Autonomous Prefecture (5.11/100,000), and Wuzhong City (5.32/100,000) in Ningxia (see Additional file 1). Compared with the annual average incidence rate of HB in 2014–2017, the annual average incidence rate of HB in 2018–2020 in some regions of the Qinghai Tibet Plateau, most regions of Xinjiang, Shaanxi, Shanxi, Henan, and Hebei in the middle, and Shandong, Beijing, and Tianjin in the east has significantly decreased. However, the incidence rate of brucellosis in eastern Tibet, central Gansu, and most parts of the Inner Mongolia Autonomous Region increased significantly (see Additional file 1).

**Fig. 1:**
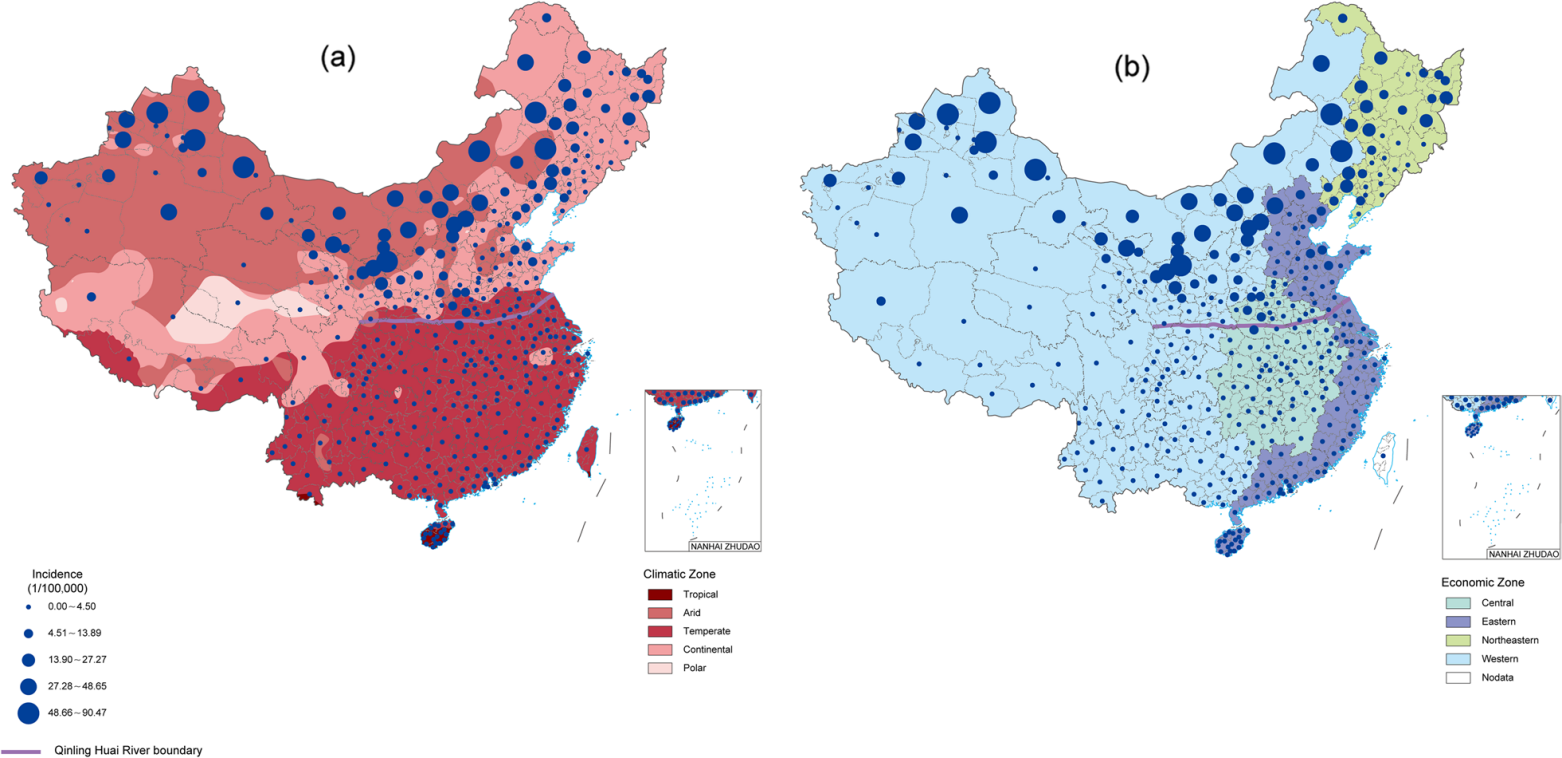
Spatial distribution of brucellosis in China by **a** climatic and **b** economic zones. Incidence rates are calculated for 2014–2020 per 100,000 people. The purple line is the Qinling Mountains-Huaihe River line divided between northern and southern China

The results show an apparent variation due to the vast size of the country and a large number of cities. The highest incidence of brucellosis in China was more than 200 times the lowest in prefecture-level cities. In terms of climate regions, from 2014 to 2020, the annual average incidence rate of HB in the arid region was the highest (1.88/100,000), followed by the continental climate zone (0.47/100,000). The incidence rate of temperature and tropical climate zones was low, at 0.048/100,000 and 0.003/100,000, respectively. In the economic belt, the annual average incidence rate of HB in the northeast economic belt is the highest at 0.68/100,000; the second highest in the western economic belt, 0.45/100,000; the incidence rate of the central economic belt and the eastern economic belt is relatively low (0.18/100,000 and 0.14/100,000, respectively; Fig. 1).

**Fig. 2:**
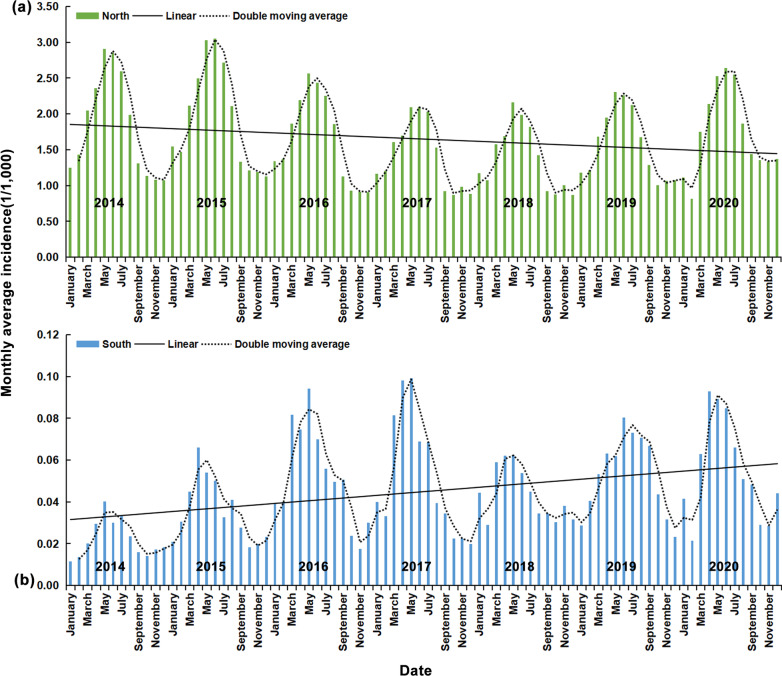
Temporal distribution of brucellosis in **a** northern and **b** southern China, divided by the Qinling Mountains-Huaihe River line. Incidence rates are calculated for 2014–2020 in units per 1 million people. The black line is the trend line

Most cases occur from March to August every year, with May being the peak point. As the incidence of brucellosis is significantly higher in northern China than in southern regions, we analyzed northern and southern China separately in our temporal distribution study. From 2014 to 2020, the incidence rate of HB in northern China was 0.65/100,000, which was much higher than that in southern China (0.02/100,000). The results are shown in Fig. 2. Northern and southern China showed opposite results. The yearly decreasing trend in the incidence of brucellosis in northern China is reflected in the results, and the incidence in southern China shows an increasing yearly trend. It should be noted that the order of magnitude of incidence rates in the North is, on average, approximately 40 times higher than that in the South, resulting in an upward trend in the South being greater than the downward trend in the North, although the slope of the trend line is the same. This is reflected in the results, as the average incidence rate in the North decreased by approximately 20% from 2014 to 2020, whereas this increased by nearly 100% in the South.

### Correlation and seasonality between brucellosis and climate

**Fig. 3:**
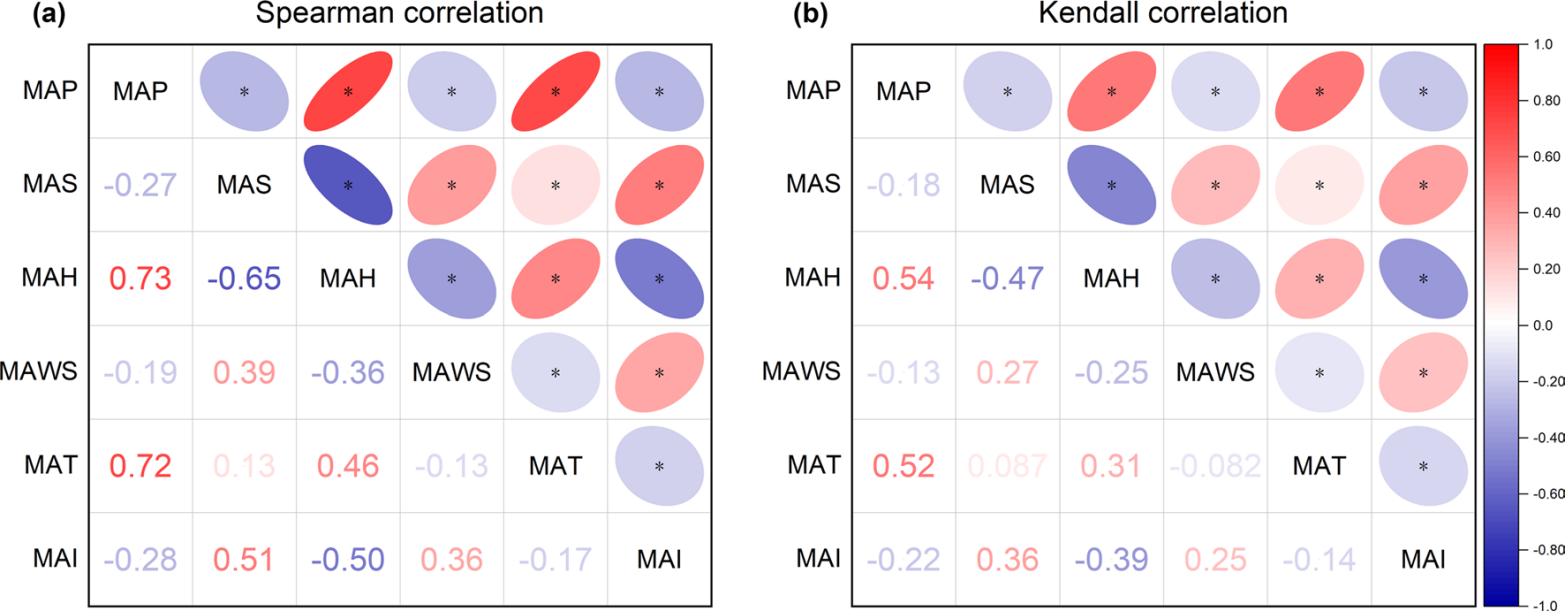
Correlation between climatic factors and incidence of brucellosis. Correlation coefficients and heat map matrices for climatic factors and incidence of brucellosis. **a** Spearman correlation, and **b** Kendall correlation. ^*^ In the heat map part of the figure represents $P<0.05,$ which indicates that the corresponding correlations are statistically significant. MAP refers to monthly average precipitation, MAS refers to monthly average sunshine, MAH refers to monthly average humidity, MAWS refers to monthly average wind speed, MAT refers to monthly average temperature and MAI refers to monthly average incidence

Before modeling, we performed a correlation analysis of the data. We found that all climatic, socioenvironmental, and brucellosis data did not satisfy the normality condition (see Additional file 1). Therefore, it was necessary to exclude the Pierce correlation in the correlation analysis and use the Spearman and Kendall correlations in the rank correlation. The results are shown in Fig. 3. Taking the MAI of brucellosis as a base, 60% of the weather data were negatively correlated and 40% were positively correlated. There was clear collinearity between the individual weather data, with some correlation coefficients being even more significant in absolute values than between them and the MAI. Compared to the other weather factors, only MAS and MAH had Spearman correlation coefficients above 0.5, which lies within the moderate correlation interval and is more significant than the other factors in the subsequent modeling analysis.

**Fig. 4:**
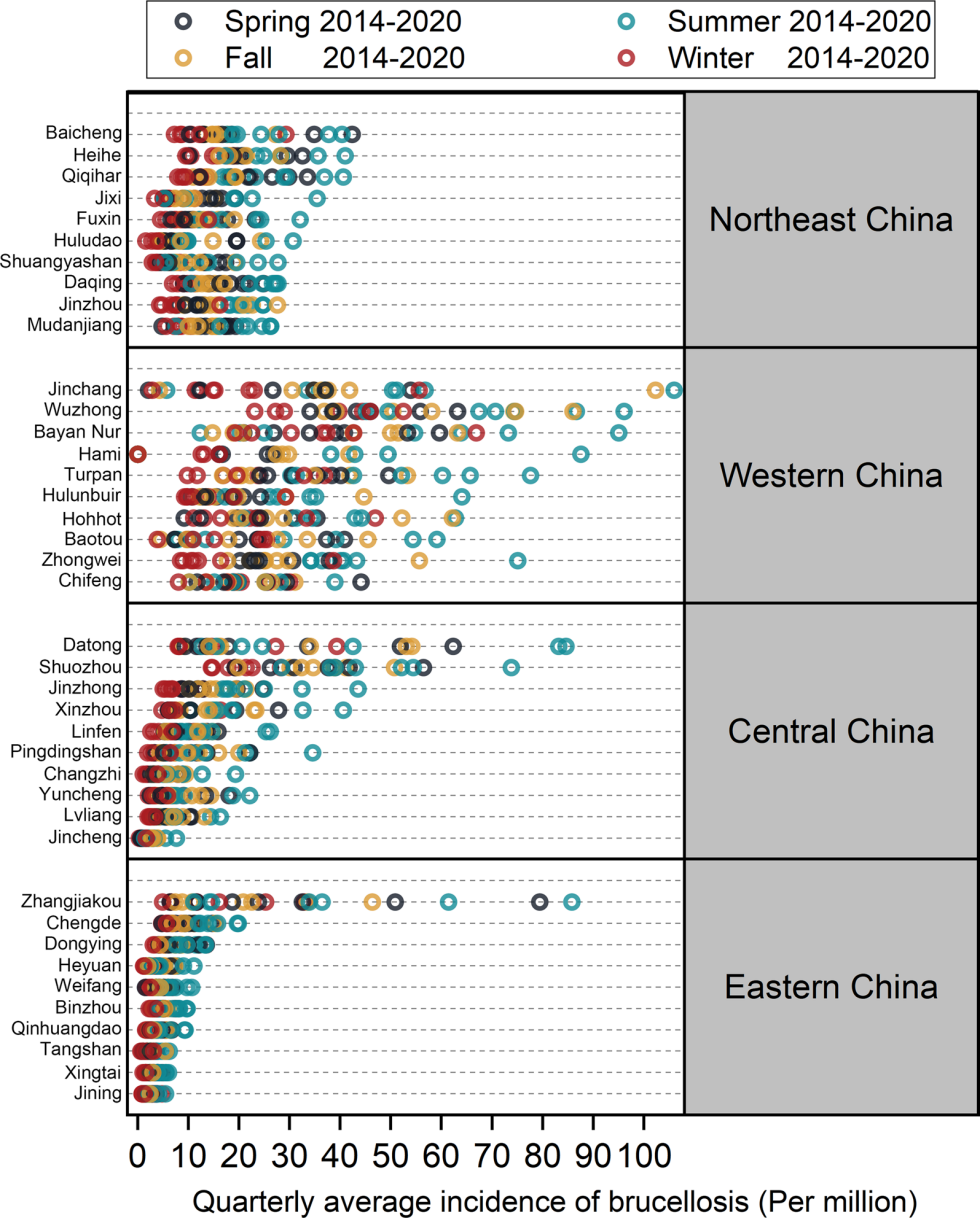
Incidence of brucellosis in different geographical regions of China by season between 2014 and 2020. The top 10 prefecture-level cities in each of the 4 geographic regions using economic division criteria for the average incidence of brucellosis are presented in the Figure colored dots represent the quarterly average incidence of brucellosis between 2014 and 2020

The incidence of brucellosis was distinctly seasonal (Fig. 4), with a high incidence in spring and summer. Overall, the average quarterly incidence rates were winter, fall, spring, and summer. The four seasons did not show a wide disparity, with a difference of approximately 30% in the incidence rate per 1 million persons. Zhangjiakou City, Hebei Province, was the top prefecture-level city in the eastern region in terms of incidence rate, far surpassing the second and subsequent cities in terms of incidence rate. Except for Zhangjiakou City, the incidence rates of the top 10 prefecture-level cities in the eastern region are slightly lower than those in the central and northern regions and far lower than those in the western region (average incidence rate per million people: 15.03 in northern China, 30.23 in western China, 13.53 in central China, 4.83 in eastern China), which is consistent with the distribution of animal husbandry in China.

### Classical statistics and SARIMAX prediction models

The weather and brucellosis data used in this study were monthly compilations, and the social and environmental data were annually compiled, all of which were time series spanning 6 years (see Fig. 2). The Kolmogorov–Smirnov, Shapiro–Wilk, and Jarque–Bera normality tests were not strictly satisfied (see Additional file 1). However, considering that the absolute value of the kurtosis was less than 10 and the absolute value of the skewness was less than 3, although the data were not absolutely normally distributed, they were basically accepted as normal distributions. Many models were built and screened based on the statistical nature and seasonality of climatic, socioenvironmental, and brucellosis data. The indicators of the models with excellent performances are shown in Table 1. The output results of these traditional statistical regression models were monthly brucellosis cases, and the input variables were climatic and socioenvironmental data.

**Table 1:** Classical statistical model performance summary

Predictive models	Adj ${R}^{2}$	Effectiveness indicator	$P$ value	Collinearity indicator
Stepwise Regression	0.489	$F$(9,13,089) = 1393.606	All variables *P* < 0.01	$D-W$ value = 0.788
Ridge Regression	0.481	$F$(8,13,090) = 1516.354	All variables *P* < 0.01	$\text{NA}$
Robust Regression	0.488	$F$(9,13,089) = 1389.404	All variables *P* < 0.01	$\text{NA}$
Quartile Regression (25%)	0.275	$Y$= − 0.406	All variables, except geographical region *P* < 0.01	$\text{NA}$
Quartile Regression (50%)	0.302	$Y$= 0.131	*P* < 0.01	$\text{NA}$
Quartile Regression (75%)	0.320	$Y$= 0.710	*P* < 0.01	$\text{NA}$
PLS regression (1 principal component)	0.525	$Q{h}^{2}$= 1.000	*P* < 0.05	$\text{PRESS}$ = 4.081
PLS regression (2 principal component)	0.544	$Q{h}^{2}$= −0.119	*P* < 0.05	$\text{PRESS}$ = 4.021
PLS regression (3 principal component)	0.552	$Q{h}^{2}$= −0.176	*P* < 0.05	$\text{PRESS}$ = 4.055
PLS regression (4 principal component)	0.555	$Q{h}^{2}$= −0.225	*P* < 0.05	$\text{PRESS}$ = 4.149

Table 1 shows that none of the traditional statistical regression models fit the data very well. Stepwise, ridge, and robust regression have similar model superiority, with ridge regression having the ability to handle linear data. The PLS regression models' GOF performs better in these models, but cannot handle data collinearity, which results in less objective results. Although they are all significant, none of the adjusted ${R}^{2}$ exceeds 0.6 and are unsuitable as predictive models.

**Fig. 5:**
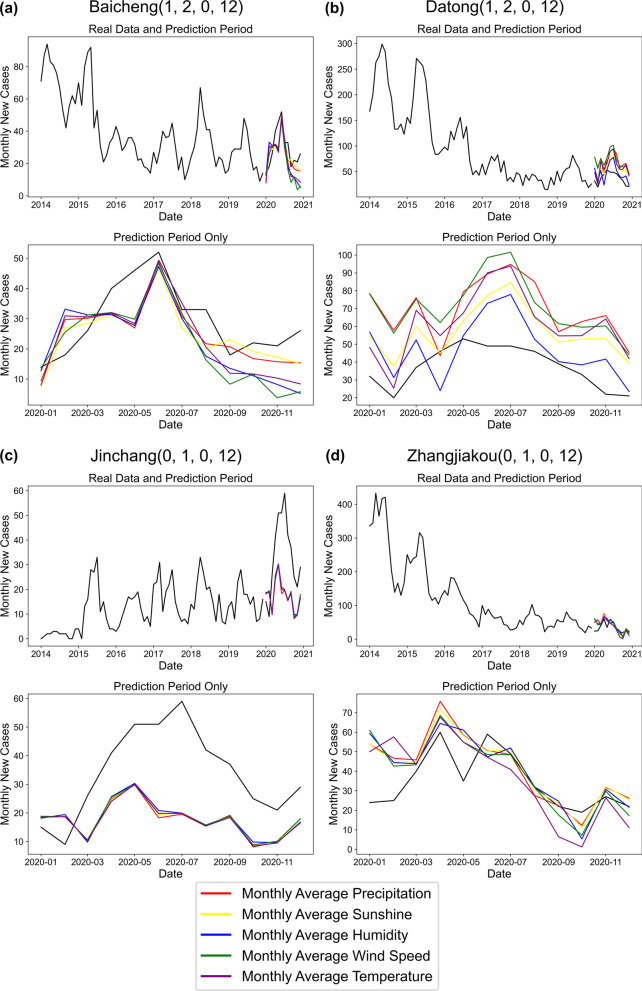
Predicted MAI of brucellosis in **a** Baicheng (in Northeast China), **b** Datong (in Central China), **c** Jinchang (in Western China), and **d** Zhangjiakou (in Eastern China) based on SARIMAX model. The four prefecture-level cities in the figure are the cities with the highest average incidence of brucellosis among the four major economic regions in China that are used as typical data for analysis. The data from 2015 to 2019 was used as the model training set, and the data from 2020 was the prediction set. The black line represents the data as a comparison in the prediction set. The colored lines represent the SARIMAX results after different climatic data are input as exogenous variables

Machine-learning models may exhibit better analytical performance than classical statistical regression models. SARIMAX is a machine learning model suitable for seasonal time-series forecasting with exogenous variables. In terms of model parameterization, we first observed the brucellosis data to determine the seasonal period, $S=12$, and found that the model $p, d, q=(\mathrm{1,1},1)$ of all input variables was the most appropriate through the automatic optimization algorithm. Subsequently, we used the seasonal decomposition sequence diagram to determine the $P, D, Q$ values of different input variables. After obtaining these results, we use AIC, BIC, and HQIC to screen the optimal model. The results after the application to the dataset used in this study are shown in Fig. 5 and Table 2.

**Table 2:** SARIMAX model performance summary

Geographical region	City	${\mathrm{SARIMAX}(\mathrm{1,1},1)(\mathrm{P},\mathrm{Q},\mathrm{D})}_{\mathrm{S}=12}$ results										
		exg	P	Q	D	AIC	BIC	HQIC	SE (exg)	Ljung-Box (LB)	Jarque–Bera (JB)	Heteroskedasticity (H)
Northeast China	Baicheng	MAP	1	2	0	424.793	434.044	428.274	0.1	0.25	5.1	0.54
		MAS	1	2	0	424.398	433.649	427.879	0.069	0.18	5.55	0.48
		MAH	1	2	0	424.896	434.146	428.377	0.448	0.23	6.2	0.53
		MAWS	1	2	0	421.492	430.743	424.973	3.19	0.15	13.98	0.54
		MAT	1	2	0	424.802	434.053	428.283	1.29	0.19	4.17	0.52
Central China	Datong	MAP	1	2	0	465.631	474.882	469.113	0.078	0.01^*^	8.46	0.5
		MAS	1	2	0	470.636	479.886	474.117	0.109	0.13	12.28	0.39
		MAH	1	2	0	468.942	478.193	472.423	0.376	0.23	13.37	0.47
		MAWS	1	2	0	471.73	480.98	475.211	4.495	0.35	16.17	0.43
		MAT	1	2	0	470.617	479.868	474.099	1.77	0.21	6.05	0.56
Western China	Jinchang	MAP	0	1	0	434.754	443.064	437.998	0.082	0.37	1.88	0.78
		MAS	0	1	0	434.841	443.152	438.085	0.025*	0.42	1.81	0.77
		MAH	0	1	0	434.49	442.801	437.734	0.112	0.38	2.07	0.65
		MAWS	0	1	0	434.885	443.195	438.129	3.825	0.39	2	0.73
		MAT	0	1	0	434.649	442.959	437.893	0.648	0.43	2.37	0.68
Eastern China	Zhangjiakou	MAP	0	1	0	723.344	731.654	726.587	0.169	0.01^*^	16.25	0.65
		MAS	0	1	0	721.686	729.996	724.93	0.255	0.03^*^	59.88	1.09
		MAH	0	1	0	716.653	724.964	719.897	0.477	0^*^	6.59	0.61
		MAWS	0	1	0	717.534	725.844	720.778	7.768	0.08	4.66	0.81
		MAT	0	1	0	723.143	731.453	726.387	0.81	0.03^*^	41.33	0.91

*$$P < 0.05$$

**Fig. 6:**
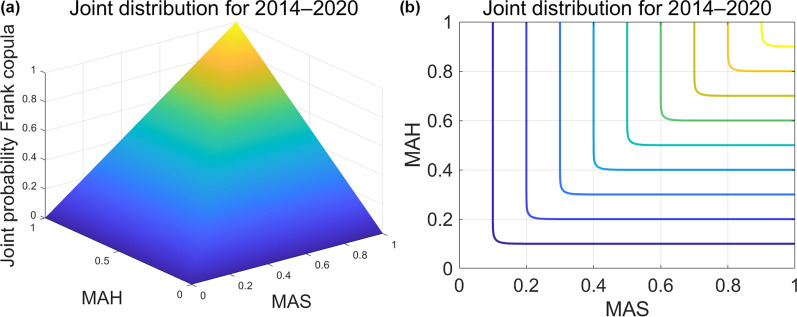
Copula **a** three-dimensional contours and **b** two- dimensional joint distribution of sunshine and humidity. The range of all axes is 0–1, representing probability values 0–100%. MAS refers to monthly average sunshine and MAH refers to monthly average humidity

The five types of climatic data entered as exogenous variables in the SARIMAX model had different effects on the prediction results. The standard errors of MAP, MAS, MAH, MAWS, and MAT in the prediction model of prefecture-level cities in the four geographical regions were 0.10725, 0.1145, 0.35325, 4.8195, and 1.1295, respectively. Among them, the performance of the prediction model for MAP, MAS, and MAH was much higher than that of the other two climatic datasets, and the accuracy of the results was higher, consistent with the findings illustrated in Fig. 6. Most of the SARIMAX prediction models that we constructed passed the white noise test and proved to be non-autocorrelated. The results of all models satisfied the normal distribution and showed no heteroscedasticity properties.

Figure 5c shows a significant deviation in the forecast results for 2020 for Jinchang, Gansu Province, as reflected in the model with all climatic data as the input. The prediction model failed due to an unexpected brucellosis pandemic in Jinchang in the summer of 2020. The average monthly incidence in July 2020 peaked in 2014–2020, to nearly twice the second place.

### Copula extreme weather model

The five types of climatic data used in this study had significant covariance, and the rank correlation coefficients between them are shown in Fig. 3. These interdependent data in extreme weather analysis can affect the accuracy and objectivity of the results. For statistical significance, we chose to have both high performances of the predictive model input variables and high-rank correlation coefficients for sunshine and humidity as climatic data for extreme weather analysis.

**Fig. 7:**
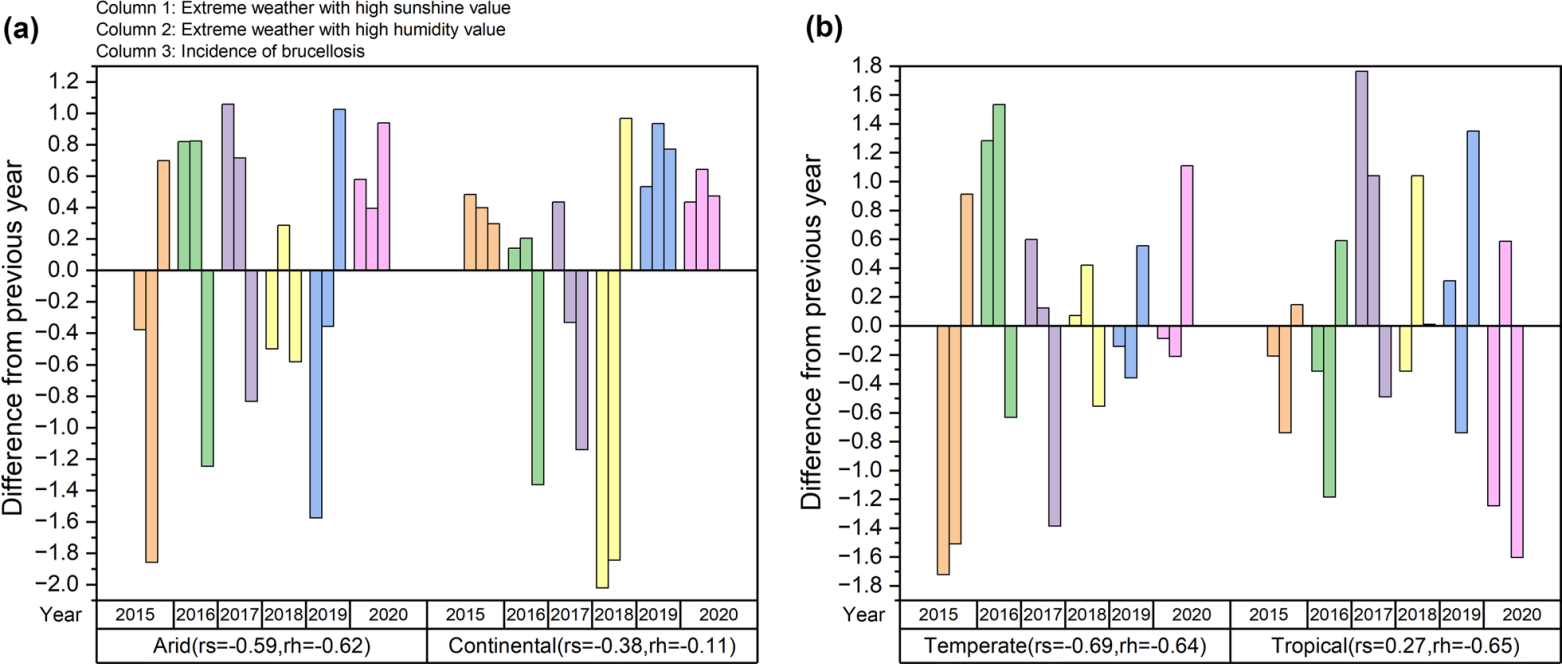
Trends in the sunshine and humidity extremes and incidence of brucellosis in **a** arid and continental, **b** temperate and tropical climatic zones after copula-processing. We normalized the differences due to order-of-magnitude gaps, which may thus lead to an unclear presentation in the figure; $rs$ represents the Pearson correlation coefficient for the year-to-year difference between sunshine and the incidence of brucellosis in the corresponding climate zone; $rh$ represents humidity. The rationale for selecting Pearson for the correlation coefficients is that the data all conform to a normal distribution (see Additional file 1) but have not been tested for statistical significance because the amount of data is too small (*n* = 6) to qualify for the test

We first performed a copula modeling analysis of the overall data, regardless of region and period, to filter out the marginal and joint distribution functions. The results are presented in Table 3 and Fig. 6. Second, we performed year-by-year modeling for the data regardless of region, and the results were not significantly different. The model performance is presented in Table 3, and the joint distribution figures are shown in Additional file 1. Based on the previous results, we performed copula modeling analysis on year-by-year climatic data from different climatic regions and explored the correlation between extreme weather and brucellosis incidence according to the quantile threshold method. The results are shown in Fig. 7.

**Table 3:** Sunshine and humidity copula model performance, 2014–2020

Climatic variables	Function	2014	2015	2016	2017	2018	2019	2020	2014–2020
Sunshine edge distribution ${R}^{2}$	Frechet	0.978	0.977	0.976	0.977	0.965	0.956	0.992	0.979
	Gumbel	0.979	0.980	0.976	0.977	0.965	0.965	0.991	0.979
	Weibull	0.995	0.997	0.994	0.997	0.991	0.986	0.997	0.997
Humidity edge distribution ${R}^{2}$	Frechet	0.899	0.886	0.887	0.915	0.890	0.917	0.894	0.906
	Gumbel	0.899	0.886	0.893	0.915	0.891	0.927	0.894	0.903
	Weibull	0.975	0.970	0.965	0.973	0.958	0.985	0.984	0.975
Copula Joint distribution function AIC (${10}^{4}$)	Clayton	−1.050	−0.943	−0.980	−1.150	−1.035	−1.271	−0.753	−7.786
	Frank	−1.486	−1.333	−1.338	−1.387	−1.365	−2.053	−1.308	−10.312
	Gumbel	−1.314	−1.253	−1.256	−1.316	−1.322	−1.913	−1.270	−9.648

Table 3 shows that the most suitable marginal distribution function for insolation and humidity is Weibull, and the copula joint distribution function is Frank. These results remain constant in all years. The performance parameters' excellent values demonstrated the copula model's positive effect in eliminating the covariance between climatic data, and the influence of other weather factors on this can be excluded in subsequent studies analyzing single-factor extreme weather.

We conducted a correlation analysis between copula-processed sunshine and humidity data classified using the quantile threshold method and the difference in the incidence of brucellosis. The results showed a significant negative correlation between sunshine and humidity extremes above the 75% percentile and a trend of variation in the incidence of brucellosis. For the sunshine data, a moderate-to-high negative correlation is reflected in the arid, temperate climatic zones. For the humidity data, a high degree of negative correlation is reflected in the arid, temperate, and tropical climatic zones.

## Discussion

Brucellosis is highly prevalent within the continental and arid climate zones of northern China, a region with vast grassland terrain, mild climate and temperature in all seasons, and a highly developed livestock industry in China. In recent years, the incidence of brucellosis in the northern region has shown an overall decreasing trend annually owing to the standardization of livestock management and the improvement of public health awareness of the population. In contrast, the incidence of brucellosis has been on the rise in southern China as sheep farming has been widely adopted. Furthermore, the increase in incidence is facilitated by a lack of experience in preventing and treating brucellosis in the southern region. The overall order of magnitude difference in the incidence rate with the North is large; however, the rising trend cannot be ignored.

The high incidence of brucellosis in spring and summer has a distinct seasonality. This is because *Brucella* is a human–animal bacterium closely associated with animal husbandry, which multiplies faster and is more biologically active in the warm season than in the cold season. In addition, cattle and sheep have a reduced rate of feeding and weight gain in spring and summer, resulting in restricted immunity and an increased risk of brucellosis. The high degree of covariance between socioeconomic and climatic data leads to very poor performance and low prediction accuracy of traditional statistical regression models in predicting the incidence of brucellosis using both socioeconomic and climatic data. The SARIMAX model improves this significantly, is suitable for seasonal time-series prediction, and is commonly used in prediction studies of various infectious diseases. We applied SARIMAX to predict the incidence of brucellosis using climatic data, and found good performance for precipitation, sunshine, and humidity.

A high degree of negative correlation was observed between the difference in year-to-year variation in sunshine and humidity in extreme weather and the incidence of brucellosis after copula processing. This is reflected by the fact that the higher the proportion of extreme weather with high sunshine or humidity values, the lower the incidence of brucellosis, which is most evident in arid and continental climatic zones. The negative correlation results generated in our study are consistent with those of other studies [30]. One possible reason is that high sunshine and high humidity extreme weather occur mostly in spring and summer, which is the time of high brucellosis incidence; however, Brucella has difficulty surviving in these extreme weather conditions.

Brucella is a human-animal bacterium that is closely related to animal husbandry. In the past decade, HB has spread throughout China. To improve the high relevance of the awareness of protecting human beings from the impact of climate change, community-based integrated monitoring of zoonosis is a promising way to reduce the impact of climate change on health. More active surveillance of brucellosis in livestock and humans in China should be coordinated and adjusted through the use of an evidence-based “one health” approach [31, 32], especially in high-risk areas and animal husbandry.

The copula model is one of the main innovations of this study. Copula functions are widely used in finance and have been used in climatic studies in recent years [33–35]; however, no study has used copula models to analyze the relationship between epidemic and climatic data. Using the copula function, we filtered and modeled the marginal and joint distributions between sunshine and humidity, which could also be applied to any other climatic data. Furthermore, we innovatively analyzed the impact of extreme weather on the incidence of brucellosis and produced scientifically valid results that other studies can corroborate. Finally, we built a wide variety of statistical regression models based on socioeconomic and climatic data to predict the incidence of brucellosis, which, together with the machine learning SARIMAX model, could provide an effective model reference for similar brucellosis prediction studies.

Our study has some limitations. Brucellosis is a zoonotic disease. The dermal, gastrointestinal, and respiratory modes of transmission result in the incidence of brucellosis in each prefecture-level city, which is influenced by population migration. However, the population migration data were not fed into our model because population migration in more than 200 prefecture-level cities would exponentially degrade the computational performance of the model and greatly increase the time and space complexity. In addition, because of the stochastic nature of the copula joint distribution function for concatenation, this study only screened out the optimal copula function for climatic data, without using the joint distribution model for further analysis or generating relevant conclusions. In addition, because of the uncertainty induced when copula joint distribution functions are connected, this study only screened the optimal copula joint distribution function applicable to climatic data, without using the joint distribution model for further analysis or generating relevant conclusions.

## Conclusions

In this study, spatial and temporal analyses revealed that HB had obvious seasonality and was highly prevalent in northern China within the arid and continental climate zone, with an annually decreasing trend. The southern region showed an increasing trend year by year, and climatic data were highly correlated with the incidence of brucellosis in China. Model comparisons indicate that traditional statistical regression models do not perform well in predicting the incidence of brucellosis using socioeconomic and climatic data, whereas machine learning SARIMAX models are more applicable. In the copula extreme weather model, we screened Weibull and Frank as the optimal marginal and joint distribution functions for analyzing climatic data and found a high degree of negative correlation between high numerical extremes of sunshine and humidity after quantile threshold classification and the difference in year-to-year variation in the incidence of brucellosis.

## Supplementary Information

**Additional file 1.** Supplementary tables and figures.

## Data Availability

The datasets used and/or analyzed during the current study are available from the corresponding author upon reasonable request.

## Abbreviations

HB: Human brucellosis
NBPCP: National Brucellosis Prevention and Control Plan
AIC: Akaike information criterion
BIC: Bayesian information criterion
HQIC: Hannan-Quin information criterion
PLS: Partial least squares
NDVI: Normalized difference vegetation index
ARIMA: Autoregressive integrated moving average
SARIMAX: Seasonal Autoregressive Integrated Moving Average X
GDP: Gross domestic product
GOF: Goodness-of-fit
MAP: Monthly average precipitation
MAS: Monthly average sunshine
MAH: Monthly average humidity
MAWS: Monthly average wind speed
MAT: Monthly average temperature
MAI: Monthly average incidence
$rs$: Pearson correlation coefficient for the year-to-year difference between sunshine and the incidence of brucellosis in the corresponding climate zone.
$rh$: Pearson correlation coefficient for the year-to-year difference between humidity and the incidence of brucellosis in the corresponding climate zone
