# Supplementary material for: Driving role of climatic and socioenvironmental factors on human brucellosis in China: machine-learning-based predictive analyses
Source: Infect Dis Poverty. 2023 Apr 12;12:36. doi: 10.1186/s40249-023-01087-y (PMC10091610; doi:10.1186/s40249-023-01087-y)
Supplement: Supplementary file 1 — Additional file 1. Supplementary tables and figures. [file 40249_2023_1087_MOESM1_ESM.docx]

Annex 1 Tests for normality of socioeconomic environmental and meteorological factors

| Data (n=3753) | Skewness | Kurtosis | Kolmogorov-Smirnov | | Shapiro-Wilk | | Jarque-Bera | |
| --- | --- | --- | --- | --- | --- | --- | --- | --- |
|  |  |  | Statistic *D* | *P* | Statistic *W* | *P* | χ^2^ | *P* |
| MAP | -0.953 | 1.163 | 0.077 | *P* < 0.01 | 0.946 | 0.000 | 777.730 | *P* < 0.01 |
| MAS | -1.273 | 3.279 | 0.079 | *P* < 0.01 | 0.931 | 0.000 | 2690.672 | *P* < 0.01 |
| MAH | -0.634 | 0.025 | 0.067 | *P* < 0.01 | 0.967 | 0.000 | 251.760 | *P* < 0.01 |
| MAWS | 0.257 | -0.292 | 0.037 | *P* < 0.01 | 0.993 | 0.000 | 54.886 | *P* < 0.01 |
| MAT | -2.216 | 6.150 | 0.179 | *P* < 0.01 | 0.762 | 0.000 | 6742.008 | *P* < 0.01 |
| TP | -0.339 | 0.414 | 0.076 | *P* < 0.01 | 0.967 | 0.000 | 98.282 | *P* < 0.01 |
| GRP | -0.876 | -1.074 | 0.303 | *P* < 0.01 | 0.734 | 0.000 | 656.287 | *P* < 0.01 |
| TVPI | -0.887 | -1.073 | 0.327 | *P* < 0.01 | 0.724 | 0.000 | 667.632 | *P* < 0.01 |
| MAI | -0.499 | 0.256 | 0.037 | *P* < 0.01 | 0.984 | 0.000 | 162.527 | *P* < 0.01 |


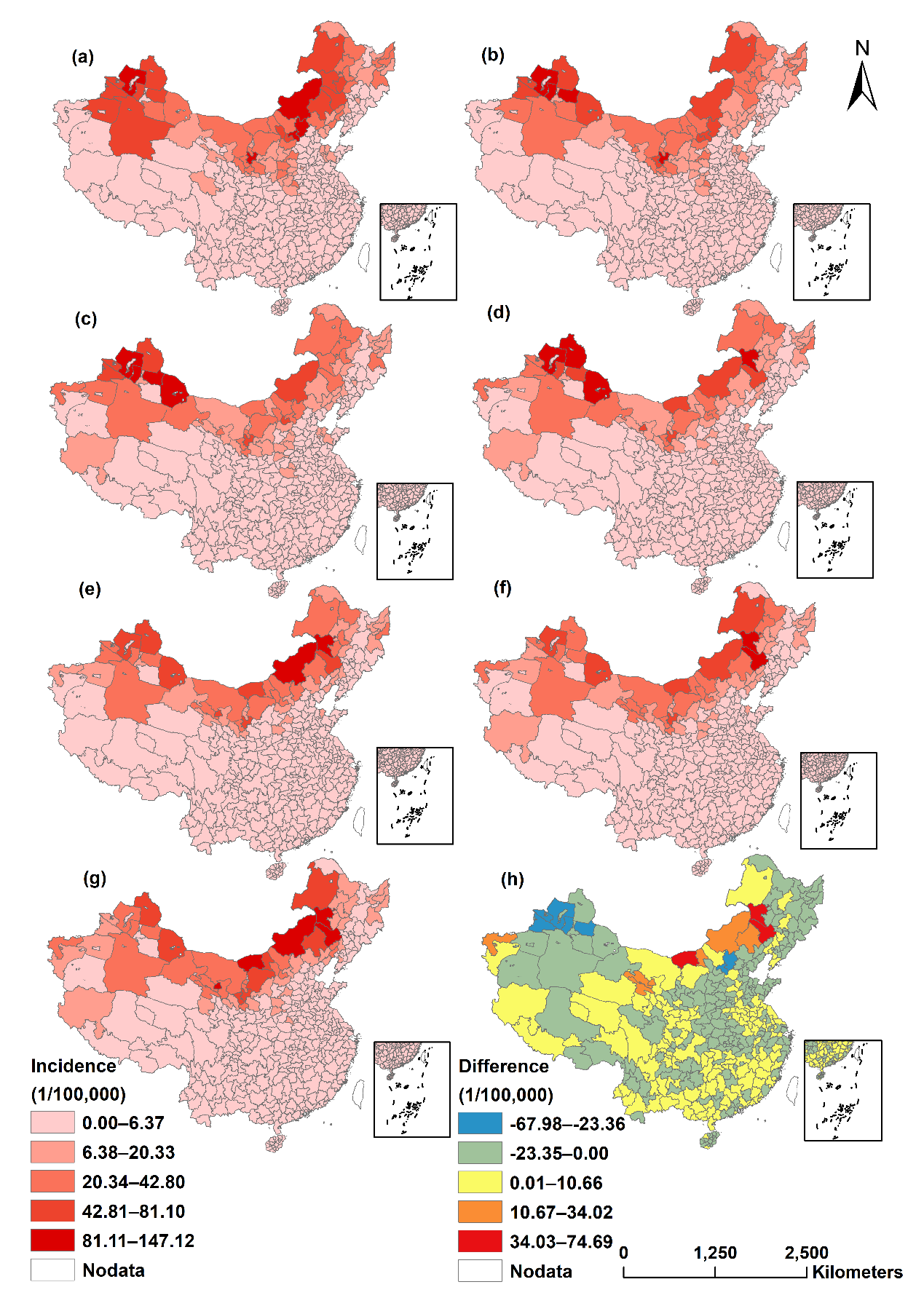


Annex 2 Annual incidence rate distribution of brucellosis in China. (a) to (g) 2014–2020; (h) Difference between the mean incidence rate from 2014 to 2017 and the mean incidence rate from 2018 to 2020.


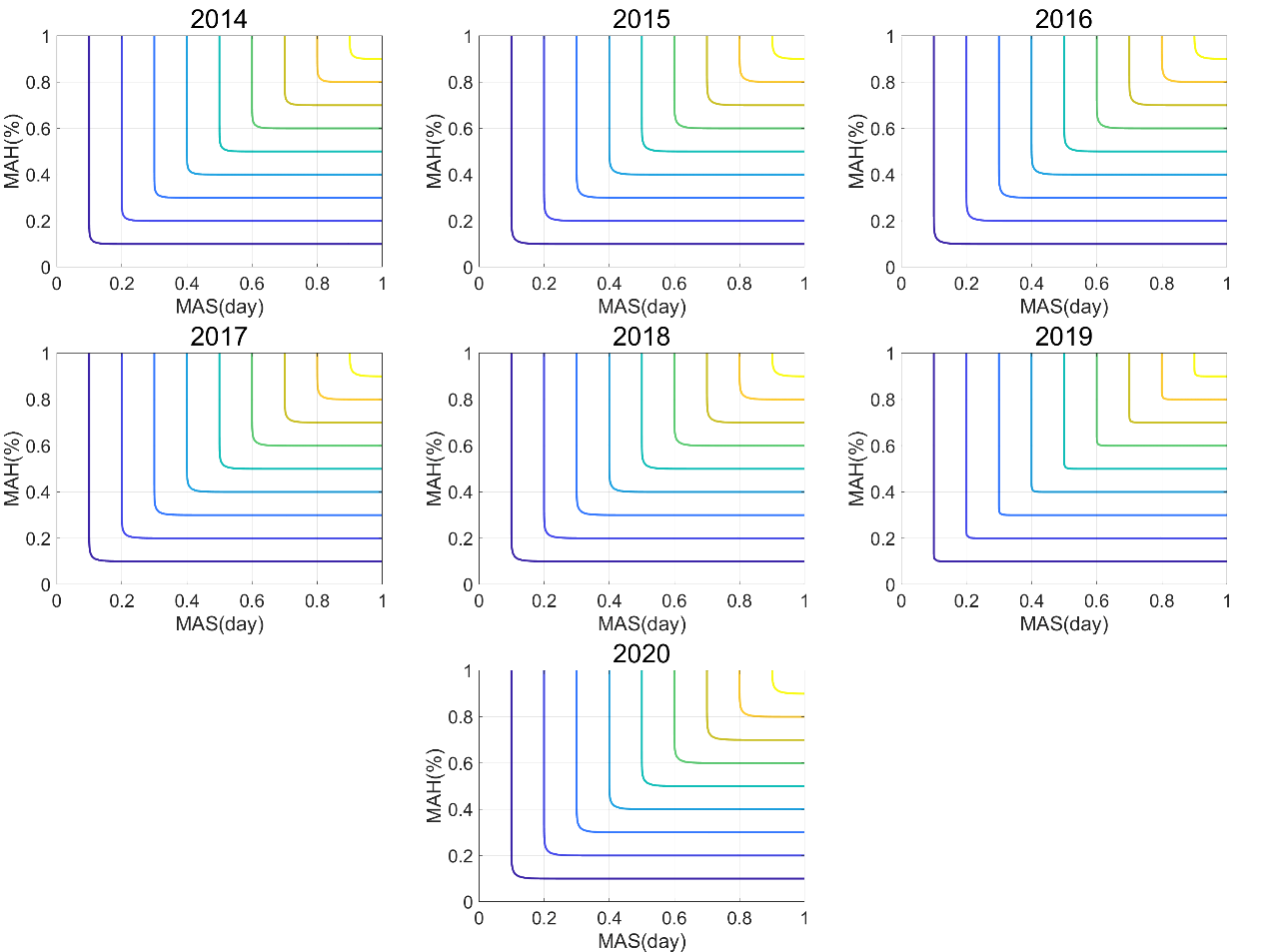


Annex 3 Two-dimensional joint distribution of sunshine and humidity (2014-2020)


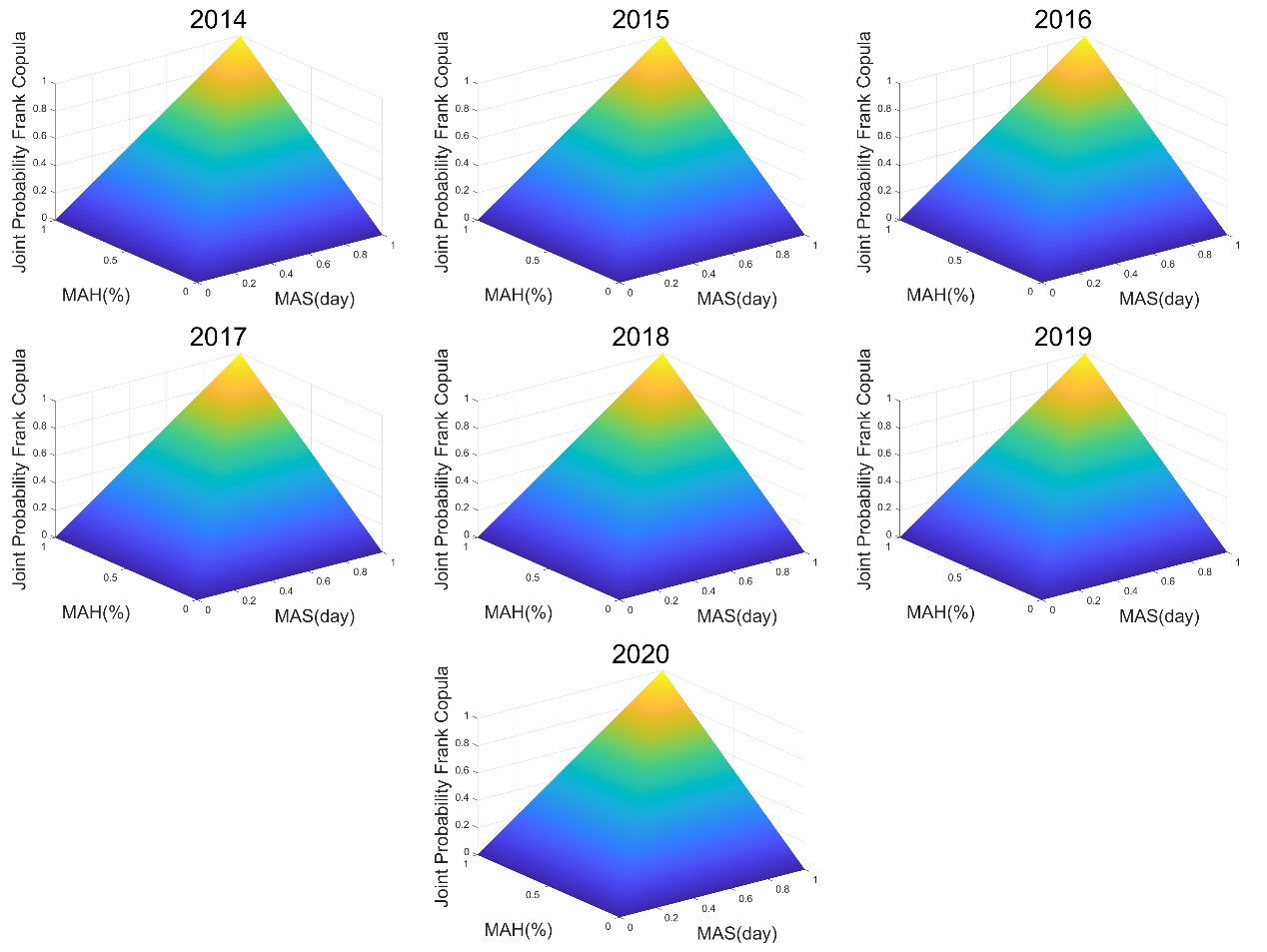


Annex 4 Three-dimensional contours of sunshine and humidity (2014-2020)

Annex 5 Test for normality of climate zone factors

| Items | *n* | Mean | Std Dev | Skewness | Kurtosis | Kolmogorov-Smirnov test | | Shapiro-Wilk test | |
| --- | --- | --- | --- | --- | --- | --- | --- | --- | --- |
|  |  |  |  |  |  | Statistic *D* | *P* | Statistic *W* | *P* |
| Arid(Weather) | 6 | 0.022 | 1.123 | -0.651 | -0.602 | 0.219 | 0.492 | 0.923 | 0.524 |
| Continent(Weather) | 6 | -0.075 | 1.824 | -2.351 | 5.592 | 0.389 | 0.005 | 0.605 | 0.001 |
| Temperate(Weather) | 6 | 0.160 | 0.828 | -0.836 | 1.890 | 0.277 | 0.157 | 0.927 | 0.558 |
| Tropical(Weather) | 6 | 0.000 | 4.225 | 1.057 | 2.245 | 0.249 | 0.289 | 0.899 | 0.365 |
| Arid(Brucellosis) | 6 | 0.880 | 2.853 | -0.142 | -2.666 | 0.257 | 0.243 | 0.857 | 0.179 |
| Continent(Brucellosis) | 6 | -0.694 | 0.748 | -0.759 | -1.745 | 0.283 | 0.139 | 0.841 | 0.133 |
| Temperate(Brucellosis) | 6 | -0.017 | 0.100 | -0.240 | -1.877 | 0.211 | 0.553 | 0.912 | 0.452 |
| Tropical(Brucellosis) | 6 | 0.001 | 0.009 | -0.474 | 0.821 | 0.171 | 0.842 | 0.980 | 0.950 |
| Arid(Weather) | 6 | -0.224 | 1.254 | -1.636 | 2.620 | 0.279 | 0.151 | 0.827 | 0.101 |
| Continent(Weather) | 6 | -0.015 | 1.838 | -1.549 | 2.539 | 0.247 | 0.299 | 0.864 | 0.202 |
| Temperate(Weather) | 6 | -0.276 | 1.175 | 0.058 | 1.305 | 0.193 | 0.688 | 0.973 | 0.913 |
| Tropical(Weather) | 6 | -0.586 | 0.986 | -0.008 | -2.738 | 0.270 | 0.184 | 0.837 | 0.124 |
